# CME ACTIVITY: Clinical Risk Factors for Severe *Clostridium difficile*–associated Disease

**DOI:** 10.3201/eid1503.070616

**Published:** 2009-03

Medscape, LLC is pleased to provide online continuing medical education (CME) for this journal article, allowing clinicians the opportunity to earn CME credit. This activity has been planned and implemented in accordance with the Essential Areas and policies of the Accreditation Council for Continuing Medical Education through the joint sponsorship of Medscape, LLC and Emerging Infectious Diseases. Medscape, LLC is accredited by the Accreditation Council for Continuing Medical Education (ACCME) to provide CME for physicians. Medscape, LLC designates this educational activity for a maximum of 0.75 *AMA PRA Category 1 Credits*™. Physicians should only claim credit commensurate with the extent of their participation in the activity. All other clinicians completing this activity will be issued a certificate of participation. To participate in this journal CME activity: (1) review the learning objectives and author disclosures; (2) study the education content; (3) take the post-test and/or complete the evaluation at **http://www.medscape.com/cme/eid**; (4) view/print certificate.

## Learning Objectives

Upon completion of this activity, participants will be able to:

- Identify the criteria used to define severe *Clostridium difficile*–associated disease (CDAD) in the current study
- Specify the prevalence of severe CDAD in the current study
- Identify the clinical risk factors for severe CDAD
- List the laboratory risk factors for severe CDAD

## Editor

**Lynne Stockton**, Technical Writer-Editor, *Emerging Infectious Diseases*. *Disclosure: Lynne Stockton has disclosed no relevant financial relationships.*

## CME Author

**Charles P. Vega, MD**, Associate Professor; Residency Director, Department of Family Medicine, University of California, Irvine. *Disclosure: Charles P. Vega, MD, has disclosed that he has served as an advisor or consultant to Novartis, Inc.*

## AUTHORS

Disclosures: Timothy J. Henrich, MD; Douglas Krakower, MD; Asaf Bitton, MD; and Deborah S. Yokoe, MD, MPH, have disclosed no relevant financial relationships.

## Earning CME Credit

To obtain credit, you should first read the journal article. After reading the article, you should be able to answer the following, related, multiple-choice questions. To complete the questions and earn continuing medical education (CME) credit, please go to **http://www.medscape.com/cme/eid**. Credit cannot be obtained for tests completed on paper, although you may use the worksheet below to keep a record of your answers. You must be a registered user on Medscape.com. If you are not registered on Medscape.com, please click on the New Users: Free Registration link on the left hand side of the website to register. Only one answer is correct for each question. Once you successfully answer all post-test questions you will be able to view and/or print your certificate. For questions regarding the content of this activity, contact the accredited provider, CME@medscape.net. For technical assistance, contact CME@webmd.net. American Medical Association’s Physician’s Recognition Award (AMA PRA) credits are accepted in the US as evidence of participation in CME activities. For further information on this award, please refer to http://www.ama-assn.org/ama/pub/category/2922.html. The AMA has determined that physicians not licensed in the US who participate in this CME activity are eligible for *AMA PRA Category 1 Credits*™. Through agreements that the AMA has made with agencies in some countries, AMA PRA credit is acceptable as evidence of participation in CME activities. If you are not licensed in the US and want to obtain an AMA PRA CME credit, please complete the questions online, print the certificate and present it to your national medical association.

### Article Title: Clinical Risk Factors for Severe *Clostridium difficile*–associated Disease

## CME Questions

1. All of the following were criteria for severe *Clostridium difficile*–associated disease (CDAD) in the current study, except:
  A. One or more intensive care unit admissions in which *C. difficile* was a major contributor
  B. Prolonged symptoms past 14 days requiring intravenous fluid replacement
  C. Colectomy or other surgery directly attributed to *C. difficile*
  D. Intestinal perforation in the setting of *C. difficile* infection
2. What was the prevalence of severe CDAD among all of the cases of CDAD in the current study?
  A. <1%
  B. 12%
  C. 29%
  D. 44%
3. Which of the following patient factors was most associated with an increased risk for severe CDAD on multivariate analysis of the current study?
  A. Age >70 years
  B. Chemotherapy use
  C. Antimicrobial use
  D. Previous hospital stay
4. All of the following laboratory factors were predictive of an increased risk for CDAD in the current study, except:
  A. White blood cell count >20,000 cells/mL
  B. Serum albumin <2.5 g/dL
  C. Creatinine >2 mg/dL
  D. Alanine aminotransferase >40 U/L

### Activity Evaluation

**Table:** 

**1. The activity supported the learning objectives.**				
Strongly Disagree				Strongly Agree
1	2	3	4	5
**2. The material was organized clearly for learning to occur.**				
Strongly Disagree				Strongly Agree
1	2	3	4	5
**3. The content learned from this activity will impact my practice.**				
Strongly Disagree				Strongly Agree
1	2	3	4	5
**4. The activity was presented objectively and free of commercial bias.**				
Strongly Disagree				Strongly Agree
1	2	3	4	5

